# 1024-pixel image matrix for chest CT – Impact on image quality of bronchial structures in phantoms and patients

**DOI:** 10.1371/journal.pone.0234644

**Published:** 2020-06-16

**Authors:** André Euler, Katharina Martini, Bettina Baessler, Matthias Eberhard, Friederike Schoeck, Hatem Alkadhi, Thomas Frauenfelder

**Affiliations:** 1 Institute of Diagnostic and Interventional Radiology, University Hospital Zurich, University of Zurich, Zurich, Switzerland; 2 Radiology Department, Groupe Hospitalier Cochin Broca Hôtel-Dieu, Université Paris Descartes, Paris, France; 3 Siemens Healthcare GmbH, Forchheim, Germany; Vanderbilt University, UNITED STATES

## Abstract

**Objectives:**

To compare objective and subjective image quality of bronchial structures between a 512-pixel and a 1024-pixel image matrix for chest CT in phantoms and in patients.

**Materials and methods:**

First, a two-size chest phantom was imaged at two radiation doses on a 192-slice CT scanner. Datasets were reconstructed with 512-, 768-, and 1024-pixel image matrices and a sharp reconstruction kernel (Bl64). Image sharpness and normalized noise power spectrum (nNPS) were quantified. Second, chest CT images of 100 patients were reconstructed with 512- and 1024-pixel matrices and two blinded readers independently assessed objective and subjective image quality. In each patient dataset, the highest number of visible bronchi was counted for each lobe of the right lung. A linear mixed effects model was applied in the phantom study and a Welch’s t-test in the patient study.

**Results:**

Objective image sharpness and image noise increased with increasing matrix size and were highest for the 1024-matrix in phantoms and patients (all, P<0.001). nNPS was comparable among the three matrices. Objective image noise was on average 16% higher for the 1024-matrix compared to the 512-matrix in patients (P<0.0001). Subjective evaluation in patients yielded improved sharpness but increased image noise for the 1024- compared to the 512-matrix (both, P<0.001). There was no significant difference between highest-order visible bronchi (P>0.07) and the overall bronchial image quality between the two matrices (P>0.22).

**Conclusion:**

Our study demonstrated superior image sharpness and higher image noise for a 1024- compared to a 512-pixel matrix, while there was no significant difference in the depiction and subjective image quality of bronchial structures for chest CT.

## Introduction

High spatial resolution is paramount to proper assessment of the submillimeter structures and pathologies inherent to the lung parenchyma and bronchial structures in computed tomography (CT) imaging [1–3]. Spatial resolution of a given CT image is technically limited by the hardware restrictions of the CT system, e.g. the detector pixel size or the focal spot size [4]. Within these given limits, spatial resolution can be modified by the reconstruction parameters selected by the user. Two main user-modifiable factors influencing spatial resolution are the reconstruction kernel and the size of the volumetric image pixel (VIP). In chest CT, fine high-contrast detail kernels are used to obtain a high spatial resolution of lung parenchyma. While the reconstruction kernel and the matrix size are typically predefined in institutional scan protocols, the size of the VIP depends on the field of view (FoV) and the number of pixels of the pixel matrix. This is of importance when reconstructing images with a large FoV covering the whole chest. Using a fixed standard image matrix of 512 x 512 pixels, the size of the VIP increases with increasing FoV and spatial resolution is consequently reduced.

Another challenge is that a standard image matrix of 512 x 512 pixels in combination with a large FoV may result in a resolution below the intrinsic resolution of a sharp, fine-detail reconstruction kernel. In such a case, the resolution of the image and the displayed structures is not limited by the CT system or the reconstruction kernel, but by the pixel matrix of the image. Details might be blurred or get lost, and the images appear pixelated. Furthermore, step artifacts become visible, especially in bony structures. If it is not desired to reduce the FoV, these effects can be mitigated by increasing the pixel matrix of the reconstructed image. Recently, one vendor has introduced larger image matrices of 768 x 768 and 1024 x 1024 pixels (Precision Matrix, Siemens Healthineers, Forchheim, Germany) as a post-processing option for CT. This post-processing option is not limited to hardware restrictions and can be applied to any CT scan.

The purpose of our study was to compare objective and subjective image quality of bronchial structures between a 512-pixel and a 1024-pixel image matrix for chest CT in phantoms and in patients.

## Materials and methods

### Part 1—Phantom study

#### Phantom and scan setup

An anthropomorphic thoracic phantom with a central bore hole and dimensions of 300 mm x 200 mm x 100 mm was used (QRM-Thorax, Quality Assurance in Radiology and Medicine (QRM), Moehrendorf, Germany). Peripheral rings of fat-equivalent material were added to emulate a medium- (outer dimensions of 350 x 250 mm) and large-sized patient (outer dimensions of 400 x 300 mm). Each of the two sizes was imaged in single-energy mode on a 192-slice CT scanner (Somatom Force, Siemens Healthineers, Forchheim, Germany) using our institutional scan protocol for chest CT with z-flying focal spot. Each size was imaged at two different radiation doses by altering the reference tube current time product (ref. mAs of 80 and 100). Details of the scan parameters are summarized in Table 1.

**Table 1:** CT scan parameters.

CT Parameter	Value
Detector collimation (mm)	192 x 0.6 (z-flying focal spot)
Tube voltage (kV)	ATVS with ref. kV of 120
Quality Reference Tube current-time product (ref. mAs)	Phantom study: 80 and 100 Patient study: 70
Gantry rotation time (sec)	0.5
Pitch	1.2

Automatic tube voltage selection (ATVS) and automatic tube current modulation (ATCM) were used.

Images were reconstructed using three different image matrices of 512 x 512, 768 x 768, and 1024 x 1024 pixels. All images were reconstructed using an advanced model-based iterative reconstruction algorithm (ADMIRE) at a strength level of three. A fine detail kernel (Bl64), a slice thickness of 1.5 mm, and an increment of 1 mm were used. The FoV was kept constant at a diameter of 316 mm for each dataset.

#### Assessment of image sharpness

**Fig 1:**
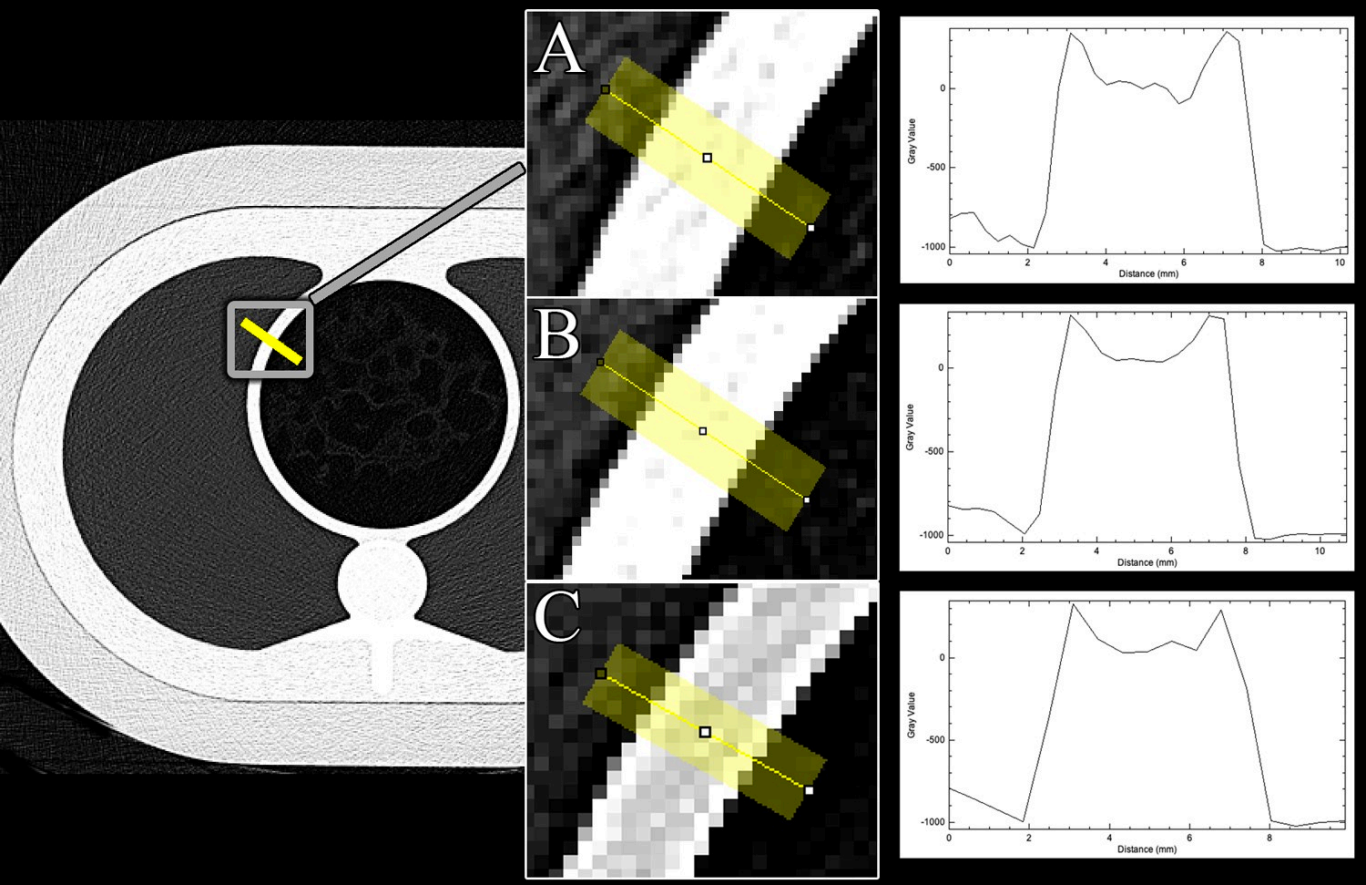
Assessment of image sharpness in the phantom. Chart shows the measurement principle of image sharpness in the phantom. CT attenuation profiles were measured along lines perpendicular to the circular central bore hole of the phantom. For each CT dataset the attenuation profile was measured at four different positions of the circular bore hole (1:30, 4:30, 7:30, 10:30 o’ clock position). Exemplary measurement profiles at the 10.5-position are shown for the 1024- (A), 768- (B), and 512-matrix, respectively. Note the increased stair-step artefacts using a 512-matrix (C).

Image sharpness was quantified according to a previously established method [5,6] by measuring the attenuation profiles along lines perpendicular to the circular central bore hole of the phantom. The attenuation profile was measured along an air-plastic-air surface transition (Fig 1) using an open-source software (Fiji [7]). For each CT dataset the attenuation profile was measured at four different positions of the circular bore hole (1:30, 4:30, 7:30, 10:30 o’ clock position). The line-width was 10 pixels and the line length 20 mm. Image sharpness was defined as the maximum steepness of the attenuation profile in CT number per mm (ΔCT_mm_) averaged across the four measurement positions.

#### Assessment of noise power spectrum

**Fig 2:**
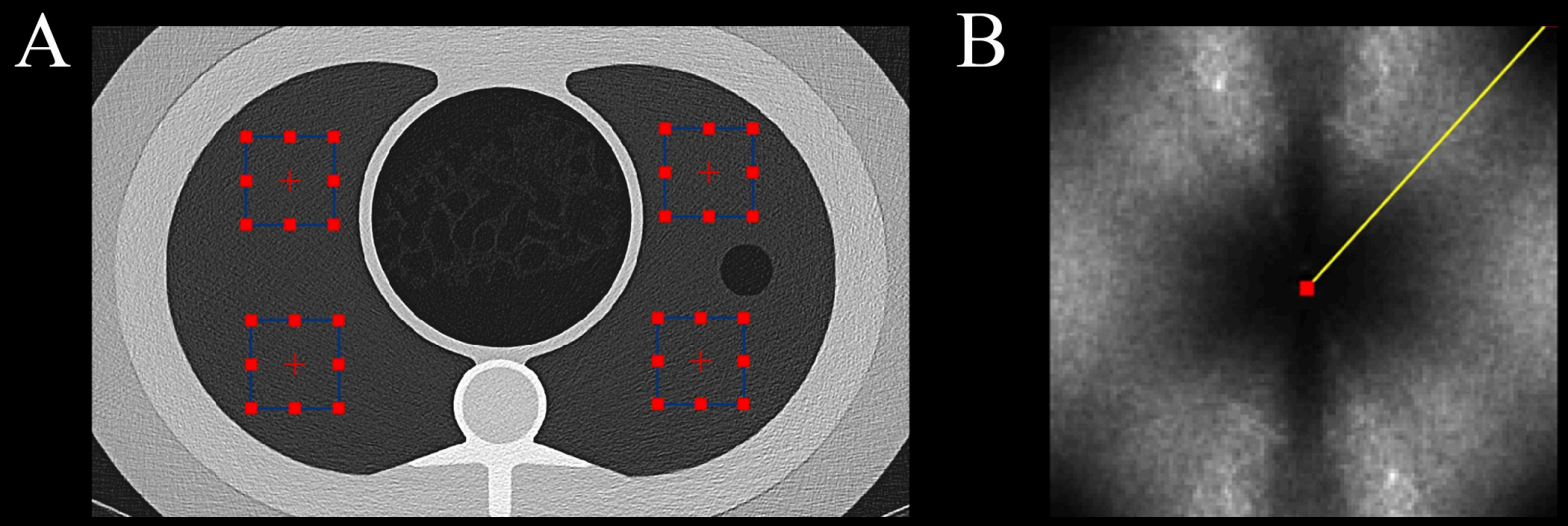
Measurement of nNPS. (A) Image demonstrating the measurement principle of the nNPS. Four square ROIs were placed at different positions in the uniformly aerated lungs of the phantom. Measurements were performed on 60 slices in each dataset. (B) 2D nNPS shapes were qualitatively assessed. The overall frequency content was compared using a 1D profile (yellow line) along the diagonal of the 2D nNPS.

The normalized noise power spectrum (nNPS) was analyzed for each dataset to investigate differences in noise texture among the reconstruction matrices. According to a previously proposed method [8–10], four 32 x 32 square regions of interest (ROIs) were placed at different positions in the uniformly aerated lungs of the phantom (Fig 2A). Measurements were performed on 60 slices in each dataset (4 ROIs per slice x 60 = 240 ROIs) using an open-source software (imQuest, Duke University, Durham, NC, USA). The overall frequency content was compared using a 1D profile along the diagonal of the resulting 2D nNPS (Fig 2B). Further, the overall 2D nNPS shapes were qualitatively assessed for the presence of noise aliasing. The average spatial frequency (F_av_) was used to compare noise texture.

### Part 2—Patient study

#### Patient population and scan protocol

This retrospective study was compliant with the Health Insurance Portability and Accountability Act (HIPPA) and approved by the local ethics committee (Cantonal Ethics Commission Zurich) under the approval number BASEC-ID 2019–01676. The requirement for study-related informed consent was waived. We retrospectively identified 100 consecutive patients who underwent a clinically indicated contrast-enhanced CT of the chest between May and July, 2019. CT was performed for the following clinical indications: follow-up of malignancy (n = 70, 70%), emphysema evaluation (n = 8, 8%), follow-up of pulmonary nodules (n = 8, 8%), pneumonia (n = 5, 5%), imaging before transplantation (n = 3, 3%), sarcoidosis (n = 3, 3%), and interstitial lung disease (n = 3, 3%).

The study population consisted of 63 men (mean age, 62.5 ± 14.6 years; median age, 66 years; age range, 27–86 years) and 37 women (mean age, 65.2 ± 14.9 years; median age, 70 years; age range, 26–92 years). Patients were imaged on a third-generation dual-source CT operated in the single-energy mode using our institutional standard chest CT protocol (Chest-STD; Table 1). Automatic tube voltage selection (CAREkV, Siemens) chose a tube voltage of 80 kVp in 2 patients, 90 kVp in 39 patients, 100 kVp in 58 patients, and 110 kVp in one patient, respectively. Mean CTDI_vol_ was 4.82 ± 2.04 mGy and mean DLP was 187.5 ± 83.5 mGy*cm. Reconstruction FoV was on average 332.1 ± 36.1 mm.

Images were reconstructed using a 512- and 1024-image matrix. Reconstruction parameters were equal to the phantom study. DICOM headers were reviewed for the diameter of reconstruction FoV of each dataset.

#### Assessment of subjective image quality and objective image noise

The overall 200 reconstructed CT datasets (100 datasets with a 512-matrix and with a 1024-matrix, respectively) were randomized and evaluated by two board-certified thoracic radiologists (both with 6 years of experience). A training session with 20 datasets was performed prior to the assessment. The readers were blinded to the reconstruction method. In lines with a prior study [11], the readers had to separately note the highest-order bronchus visible in the upper, middle, and lower lobe of the right lung, respectively. Furthermore, the sharpness of the third- or fourth-order bronchi was evaluated using a 5-point Likert-scale (5 = excellent; 4 = good; 3 = moderate; 2 = poor; 1 = blurry, non-diagnostic). Image noise and bronchial image quality were scored using a 5-point scale (5 = excellent; 4 = good; 3 = moderate, but sufficient for diagnosis; 2 = poor, diagnostic confidence substantially reduced; 1 = very poor, non-diagnostic). Of note, higher image noise resulted in a lower score. Objective image noise was measured as the standard deviation of the CT number (in Hounsfield units) obtained from a ROI individually placed by each reader in the air-filled trachea immediately cranial of the carina. The trachea was chosen because it is typically located at the patient center.

### Statistical analysis

In the phantom study, a linear mixed effects model was used to determine the effect of matrix size, phantom size, and radiation dose (reference mAs) on the image sharpness. In the patient study, the highest-order visible bronchus, image sharpness, noise, and diagnostic quality were compared between the 512- and 1024-matrix using the Welch t-test for paired samples. To account for the subjective nature of the grading task as well as to account for potential discrepancies in image quality perception between the readers, analysis was performed for each reader individually. Inter-reader agreement was tested using the intraclass correlation coefficient (ICC) for data on the interval scale and Kendall coefficient of concordance W (values 0.5–0.8, good agreement; values > 0.8, excellent agreement [12]) for data on the ordinal scale. Statistical significance was defined as P<0.05.

## Results

### Part 1—Phantom study

#### Assessment of image sharpness

**Fig 3:**
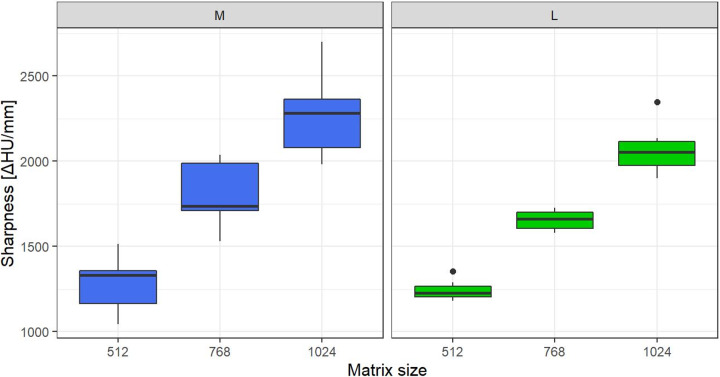
Results of image sharpness. Box plots show comparisons of image sharpness (as CT number increase per mm) among the three matrix sizes for the medium (M) and large (L) phantom sizes. Data was averaged for both radiation doses. Sharpness increased with matrix size for both sizes.

The pixel size was 0.617 mm, 0.418 mm, 0.309 mm for the 512-, 768-, and 1024-matrix, respectively. The linear mixed effects model identified image sharpness (represented by the attenuation profile as the increase in CT number per mm (ΔCT_mm_)) to be significantly influenced by matrix size (effect size = 0.84, P<0.001) and to a lesser extent by phantom size (effect size = 0.03, P<0.007). There was no significant influence of radiation dose (effect size = 0.00, P = 0.71) on ΔCT_mm_. For both phantom sizes, ΔCT_mm_ increased with image matrix size (all P<0.001; see Fig 3).

#### Assessment of noise power spectrum

**Fig 4:**
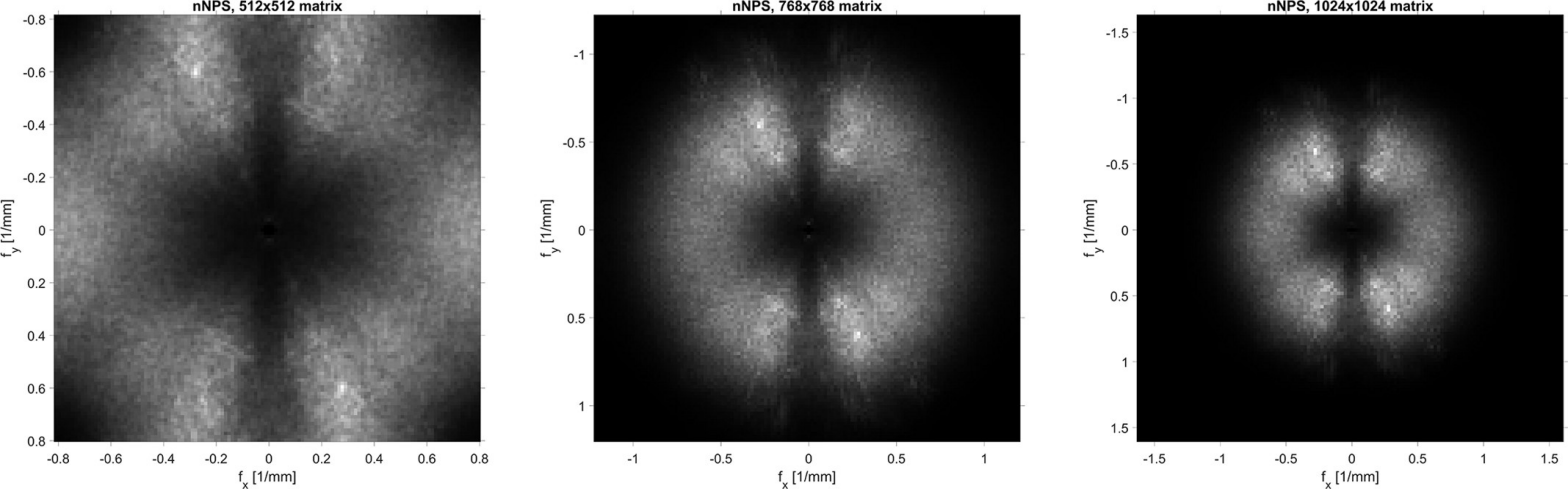
Shape of the 2D nNPS. Comparison of the shape of the 2D nNPS among all three matrix sizes. The shape for the 512-matrix demonstrated distortion, indicating aliasing effects. The diagonal 1D profile of the 2D nNPS was not affected by distortion and was therefore used as a non-biased metric to compare the noise texture.

**Fig 5:**
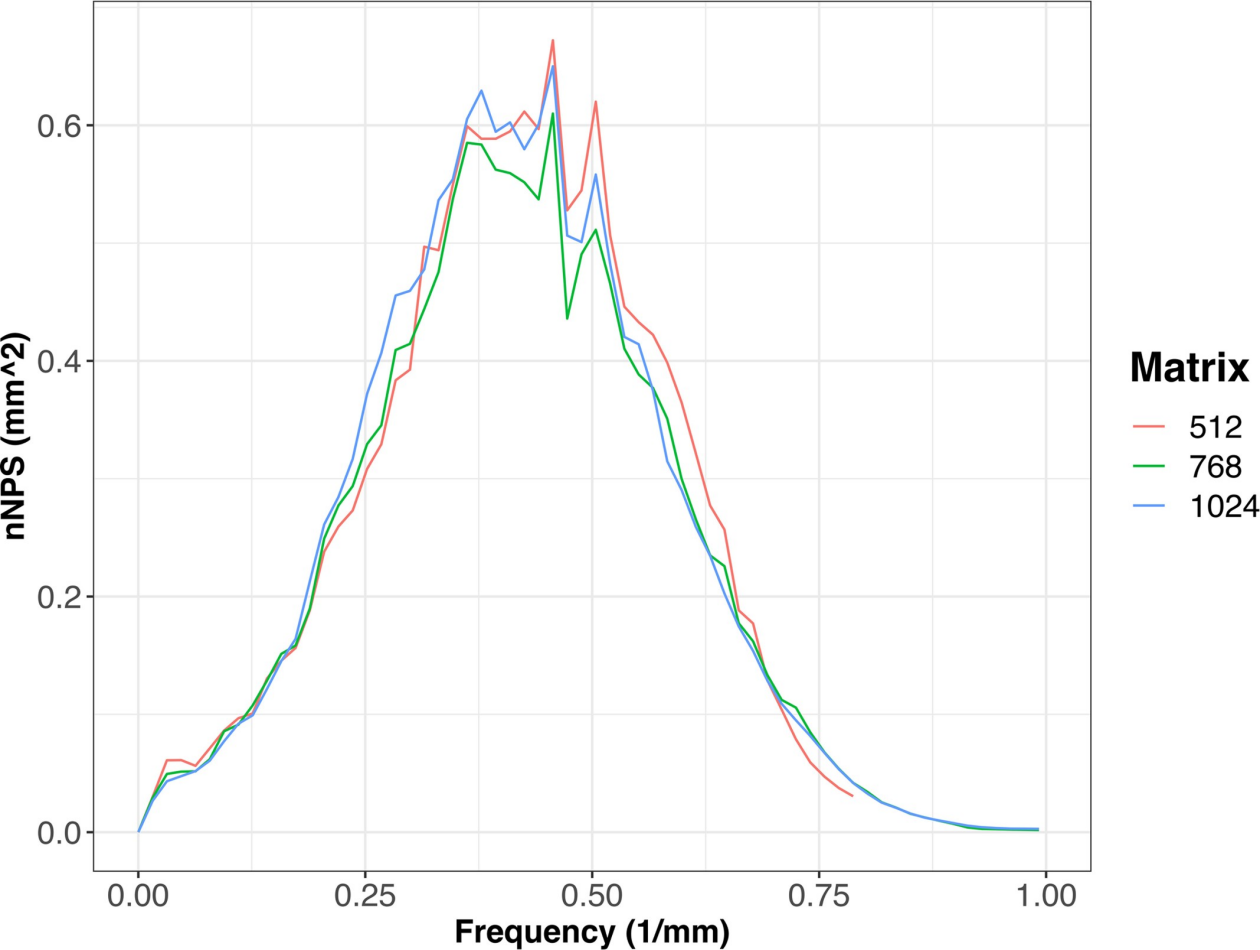
Comparison of noise texture. Graphs demonstrate similarity of the overall frequency content of the diagonal 1D profile of the 2D nNPS for each image matrix, indicating similar image noise texture.

Qualitative assessment of the 2D nNPS shapes revealed distortion of the overall shape for the 512- matrix, indicating aliasing effects (Fig 4). This distortion was not observed for the other matrix sizes as well as in the 1D diagonal profiles of the 2D nNPS. Comparison of the 1D diagonal profiles revealed no differences in noise texture among the three matrix sizes (see Fig 5). F_av_ was 0.421, 0.420, and 0.422 for the 512, 768, and 1024-matrix, respectively.

### Part 2—Patient study

Objective image noise was significantly higher for the 1024-matrix compared to the 512-matrix for both readers (both P<0.0001; see Table 2). Inter-reader agreement for objective image noise was excellent (ICC = 0.826). Image noise was on average about 16% higher for the 1024-matrix.

**Table 2:** Results of objective and subjective image quality evaluation.

	Reader 1			Reader 2			Interreader Agreement
Matrix Size	512	1024	P-value	512	1024	P-value	
HOB Upper Lobe	5.4±0.8	5.5±0.9	0.33	4.8±0.8	4.9±0.8	0.30	ICC = 0.00
HOB Middle Lobe	4.9±0.6	5.0±0.5	0.07	4.7±0.9	4.9±0.8	0.24	ICC = 0.28
HOB Lower Lobe	6.6±0.9	6.6±1.1	0.63	4.7±0.7	4.8±0.7	0.12	ICC = 0.00
Bronchus Sharpness	3.0±0.4	3.8±0.4	<0.0001*	3.2±0.7	3.5±0.6	0.007*	W = 0.665
Subjective Noise	3.4±0.5	3.0±0.3	<0.0001*	3.4±0.5	3.2±0.6	0.03*	W = 0.654
Objective Noise (HU)	68±9	79±12	<0.0001*	68±12	77±11	<0.0001*	ICC = 0.826
Diagnostic Quality	3.9±0.3	3.9±0.3	0.22	3.6±0.6	3.7±0.6	0.47	W = 0.635

HOB = Highest-order bronchus. Note that a lower subjective image noise score refers to increased image noise. Data is given as means ± standard deviation. P-value refers to the comparison between 512- and 1024-matrix. Significant p-values are indicated with

*. Intraclass correlation coefficient (ICC) was performed for data on the interval scale. Kendall’s coefficient of concordance (W) was used for data on the ordinal scale.

Analysis of subjective image quality demonstrated similar tendencies for both readers (Table 2). There was no significant difference in the highest-order bronchus visible in the upper, middle, and lower lobe of the right lung, respectively between the 1024- and 512-matrix (P-range, 0.068–0.63). Inter-reader agreement for the visibility of the highest-order bronchi showed poor agreement (ICC-range, 0–0.28). Image sharpness was scored significantly higher and image noise significantly lower for the 1024-matrix compared to the 512-matrix by both readers (all P<0.001).

**Fig 6:**
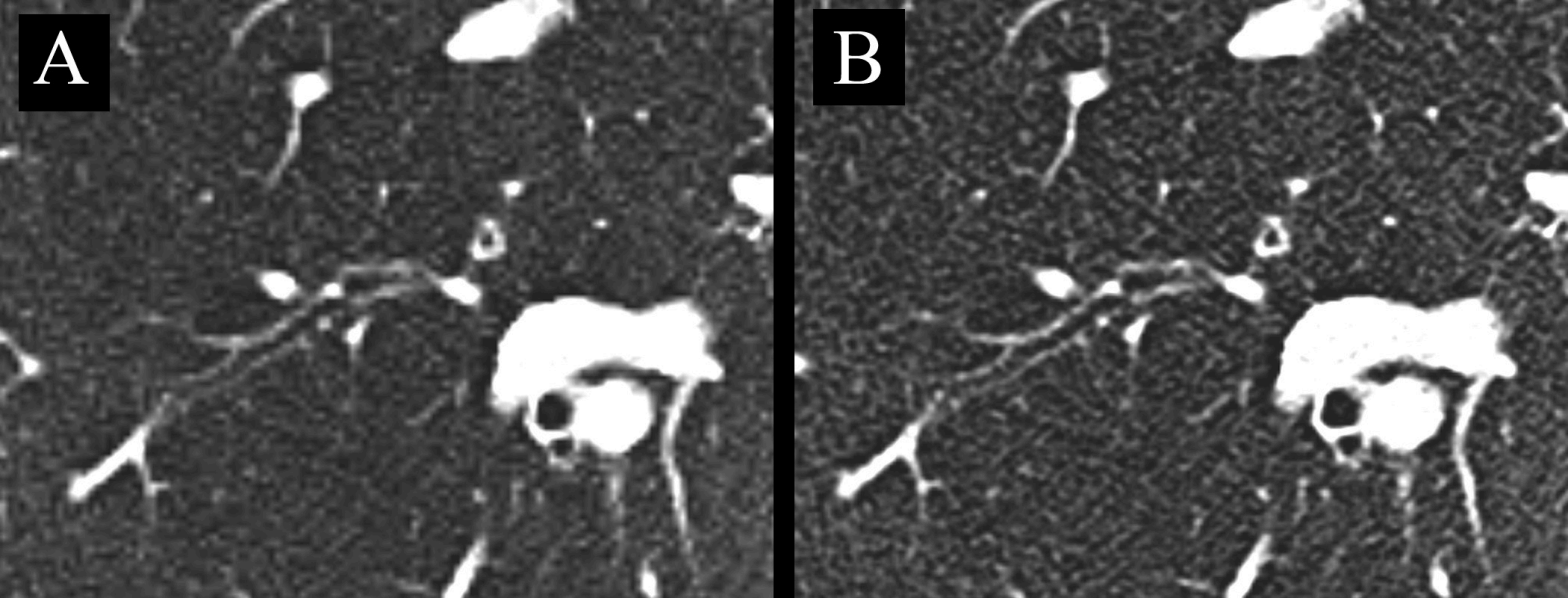
Image example of an axial CT slice of the same patient reconstructed with a 512-matrix (A) and 1024-matrix (B). Note the improved sharpness of the bronchial wall but increased image noise in B compared to A. Even though step artifacts are present in A, readers gave similar ratings for overall image quality of bronchial structures.

**Fig 7:**
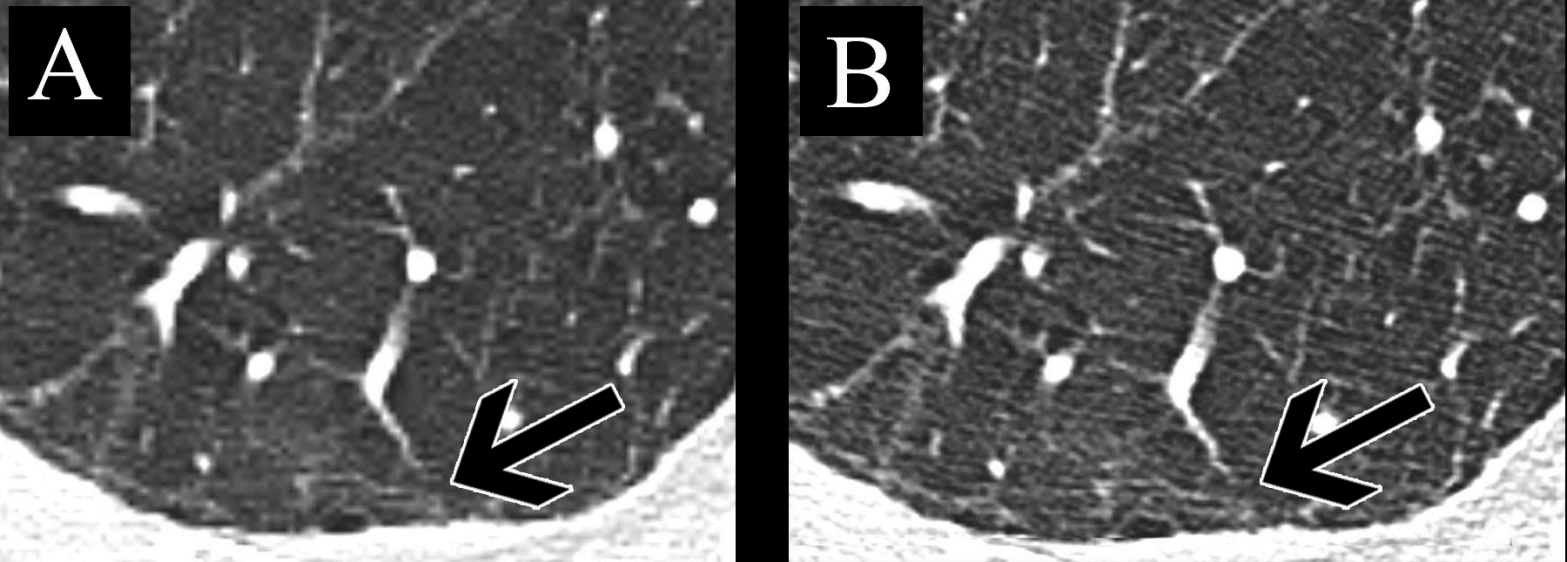
Image examples of a 67-year-old man imaged for interstitial pneumopathy. Axial CT slice reconstructed with a 512-matrix (A) and 1024-matrix (B). Fibrotic changes (black arrow) appear to be sharper in B compared to A. Objective and subjective image noise are inferior in B.

For both readers, there was no significant difference in bronchial image quality between the two matrices (both P>0.17). Inter-reader agreements for subjective sharpness, subjective image noise, and image quality were good (Kendall W-range, 0.635–0.665; all P<0.006). Image examples are provided in Figs 6 and 7.

## Discussion

Our phantom and patient studies demonstrated improved image sharpness, higher image noise, similar numeric depiction of high-order bronchi and similar subjective image quality of bronchial structures of a 1024-image matrix compared to a traditional 512-matrix for chest CT.

The motivation of our study was to assess whether image quality benefits from image matrix sizes above 512 pixels in the case of chest CT studies, where a large field of view (FoV) is typically combined with sharp, fine-detailed reconstruction kernels.

As discussed earlier, a default image matrix of 512 pixels may limit the spatial resolution, depending on the resolution of the reconstruction kernel and the size of the FoV. At a given number of pixels, the VIP is increasing with larger FoV. The VIP determines the resolution of the pixel matrix and in case it is smaller than the resolution of the reconstruction kernel, it has the same effect as a low-pass filter: the image is smoothed and consequently image noise is diminished. Spatial resolution is lost and step artifacts can become apparent especially at high-contrast tissue borders. These effects are increasing with increasing size of the VIP and increasing sharpness of the reconstruction kernel. This was validated in our study by demonstrating that objective and subjective image sharpness was significantly improved by using a 1024-pixel matrix instead of a 512-pixel matrix. In our case with a sharp kernel and a large FoV of more than 300 mm, image noise was suppressed with the 512-pixel matrix because of the smoothing effect. Consequently, increased image noise was observed using the 1024-pixel matrix.

In addition to traditional measurements of image noise, we analyzed the nNPS to compare the noise texture among the matrix sizes. We observed a distortion of the shape of the 2D nNPS for the 512-matrix. For the 512-matrix, the sampling rate (i.e. the number of pixels per mm) was not high enough to represent the high frequency noise. Those high frequency noise components were then aliased and therefore transformed to low frequency components which lead to a distortion of the 2D nNPS shape. Because this distortion did not occur for the 1D diagonal profile of the 2D nNPS, we used this profile as an unbiased surrogate to compare noise texture. The 1D diagonal profile demonstrated comparable overall noise frequency content among all three matrix sizes indicating that the noise texture is independent of matrix size and mainly influenced by the reconstruction kernel.

Multiple studies have assessed the impact of a 1024-matrix in combination with a specific ultra-high-resolution CT (U-HRCT) equipped with a smaller detector element [13–16]. A former study found improved image quality using U-HRCT with a 1024-matrix and a small FoV of 100 mm compared to a traditional CT with a 512-matrix and a FoV of 350 mm [13]. In contrast to this study, we decided to compare image quality at the same FoV to avoid effects induced by the size of the FoV. For our study, we have purposely chosen a large FoV to obtain a VIP size where the smoothing effect of the 512-matrix compared to the 1024-matrix is relevant, i.e. where the resolution of the image is not limited by the reconstruction kernel but by the size of the VIP. Another recent study has reported improved airway evaluation using a 1024-image matrix in a photon-counting CT compared to a 512- and 1024-matrix in a traditional system equipped with an energy-integrating detector (EID) in 22 patients [11]. This finding is explained by the hardware-dependent higher spatial resolution of photon-counting CT systems compared to EID-systems. For the EID-systems, the authors found significantly higher number of higher-order bronchi using the 1024-matrix compared to the 512-matrix only for the middle lobe. In contrast, our study did not show significant differences between the two matrices for the upper-, middle, and lower lobes. In their study, the authors reported similar image noise between the 512- and 1024-matrix in the EID-system while image noise in our study increased on average by 15% in the medium phantom, by 21% in the large phantom, and by 16% in the patient cohort using the 1024-matrix.

This discrepancy can be attributed to the use of a softer reconstruction kernel (Bv46) in the cited study, compared to the sharp, fine-detail reconstruction kernel used in our study (Bl64). In this situation, resolution was limited by the resolution of the softer reconstruction kernel. Consequently, they did not observe the smoothing effect related to the small 512-pixel matrix and image noise was identical to the 1024-matrix.

The following limitations of our study merit consideration. First, our chest phantom did not include lung parenchyma. Therefore, measurements of image sharpness could not be performed along fine structures as for example subpleural reticulations. Second, the phantom did not allow for the measurement of spatial resolution as modulation transfer function (MTF) or task-based transfer function (TTF). Instead, we used a previously established method assessing the attenuation profile along a surface transition. Future studies could focus in these parameters. Third, despite a training session, the assessment of the highest-order visible bronchi showed only poor agreement between the two readers. We attribute this to the subjective nature of the task as well as to potential discrepancies in image quality perception between the readers. Therefore, we analyzed subjective image quality individually for each reader. Fourth, we did not compare diagnostic accuracy, e.g. for the diagnosis of interstitial pneumopathies, between the two matrices. As demonstrated in our image examples, the superior spatial resolution provided by the 1024-matrix could, however, be beneficial in the assessment of interstitial lung disease or early fibrotic changes which typically manifest as very thin and fine subpleural reticulations. Lastly, we did not assess the impact of the 1024-matrix on the clinical workflow. While the higher number of pixels potentially allows for enlarging images without blurring and minimizes step artifacts, the additional image information may increase reading time, data transfer and data storage needs.

In conclusion, our study demonstrated superior image sharpness and higher image noise for a 1024- compared to a 512-pixel matrix, while there was no significant difference in the depiction and subjective image quality of bronchial structures for chest CT.

Further studies are required to assess the impact of the 1024-matrix on the depiction and diagnostic accuracy of fine reticular subpleural changes in interstitial lung disease.

## Supporting information

S1 File Results nNPSDiagonalProfiles. (CSV)

S2 File Results objective sharpness assessment. (XLSX)

S3 File Results bronchi depiction and subjective image quality. (XLSX)

## References

[1] Balestro E, Cocconcelli E, Giraudo C, Polverosi R, Biondini D, Lacedonia D, et al High-Resolution CT Change over Time in Patients with Idiopathic Pulmonary Fibrosis on Antifibrotic Treatment. J Clin Med. 2019 9 15;8(9). 10.3390/jcm8091469 PMC6780456 31540181

[2] Suzuki A, Sakamoto S, Kurosaki A, Kurihara Y, Satoh K, Usui Y, et al Chest High-Resolution CT Findings of Microscopic Polyangiitis: A Japanese First Nationwide Prospective Cohort Study. AJR Am J Roentgenol. 2019 4 11;1–11. 10.2214/AJR.18.20967 30973774

[3] Kim GHJ, Weigt SS, Belperio JA, Brown MS, Shi Y, Lai JH, et al Prediction of idiopathic pulmonary fibrosis progression using early quantitative changes on CT imaging for a short term of clinical 18-24-month follow-ups. Eur Radiol. 2020 2;30(2):726–34. 10.1007/s00330-019-06402-6 31451973

[4] Kawashima H, Ichikawa K, Takata T, Nagata H, Hoshika M, Akagi N. Technical Note: Performance comparison of ultra-high-resolution scan modes of two clinical computed tomography systems. Med Phys. 2020 2;47(2):488–97. 10.1002/mp.13949 31808550

[5] von Spiczak J, Morsbach F, Winklhofer S, Frauenfelder T, Leschka S, Flohr T, et al Coronary artery stent imaging with CT using an integrated electronics detector and iterative reconstructions: first in vitro experience. J Cardiovasc Comput Tomogr. 2013 8;7(4):215–22. 10.1016/j.jcct.2013.08.003 24148775

[6] von Spiczak J, Mannil M, Peters B, Hickethier T, Baer M, Henning A, et al Photon Counting Computed Tomography With Dedicated Sharp Convolution Kernels: Tapping the Potential of a New Technology for Stent Imaging. Invest Radiol. 2018;53(8):486–94. 10.1097/RLI.0000000000000485 29794949

[7] Schindelin J, Arganda-Carreras I, Frise E, Kaynig V, Longair M, Pietzsch T, et al Fiji: an open-source platform for biological-image analysis. Nat Methods. 2012 6 28;9(7):676–82. 10.1038/nmeth.2019 22743772 PMC3855844

[8] Solomon JB, Christianson O, Samei E. Quantitative comparison of noise texture across CT scanners from different manufacturers. Med Phys. 2012 10;39(10):6048–55. 10.1118/1.4752209 23039643

[9] Solomon J, Wilson J, Samei E. Characteristic image quality of a third generation dual-source MDCT scanner: Noise, resolution, and detectability. Med Phys. 2015 8;42(8):4941–53. 10.1118/1.4923172 26233220

[10] Euler A, Solomon J, Marin D, Nelson RC, Samei E. A Third-Generation Adaptive Statistical Iterative Reconstruction Technique: Phantom Study of Image Noise, Spatial Resolution, Lesion Detectability, and Dose Reduction Potential. AJR Am J Roentgenol. 2018 6;210(6):1301–8. 10.2214/AJR.17.19102 29702019

[11] Bartlett DJ, Koo CW, Bartholmai BJ, Rajendran K, Weaver JM, Halaweish AF, et al High-Resolution Chest Computed Tomography Imaging of the Lungs: Impact of 1024 Matrix Reconstruction and Photon-Counting Detector Computed Tomography. Invest Radiol. 2019;54(3):129–37. 10.1097/RLI.0000000000000524 30461437 PMC6363870

[12] Lin Z, Zhang X, Guo L, Wang K, Jiang Y, Hu X, et al Clinical feasibility study of 3D intracranial magnetic resonance angiography using compressed sensing. J Magn Reson Imaging. 2019 12;50(6):1843–51. 10.1002/jmri.26752 30980468

[13] Zhu H, Zhang L, Wang Y, Hamal P, You X, Mao H, et al Improved image quality and diagnostic potential using ultra-high-resolution computed tomography of the lung with small scan FOV: A prospective study. PloS One. 2017;12(2):e0172688 10.1371/journal.pone.0172688 28231320 PMC5322956

[14] Yanagawa M, Hata A, Honda O, Kikuchi N, Miyata T, Uranishi A, et al Subjective and objective comparisons of image quality between ultra-high-resolution CT and conventional area detector CT in phantoms and cadaveric human lungs. Eur Radiol. 2018 12;28(12):5060–8. 10.1007/s00330-018-5491-2 29845337 PMC6223853

[15] Tanabe N, Oguma T, Sato S, Kubo T, Kozawa S, Shima H, et al Quantitative measurement of airway dimensions using ultra-high resolution computed tomography. Respir Investig. 2018 11;56(6):489–96. 10.1016/j.resinv.2018.07.008 30392536

[16] Hata A, Yanagawa M, Honda O, Kikuchi N, Miyata T, Tsukagoshi S, et al Effect of Matrix Size on the Image Quality of Ultra-high-resolution CT of the Lung: Comparison of 512 × 512, 1024 × 1024, and 2048 × 2048. Acad Radiol. 2018;25(7):869–76. 10.1016/j.acra.2017.11.017 29373211

